# Common Mortality Factors of Woodwasp Larvae in Three Northeastern United States Host Species

**DOI:** 10.1673/031.012.8301

**Published:** 2012-07-18

**Authors:** Kelley E. Zylstra, Victor C. Mastro

**Affiliations:** ^1^ U. S. Department of Agriculture, APHIS, PPQ, 374 Northern Lights Dr. North Syracuse NY 13212. Kelley.E.Zylstra@APHIS.USDA.gov; ^2^ U. S. Department of Agriculture, APHIS, PPQ, 1398 West Truck Rd., Buzzards Bay, MA 02542. Vic.Mastro@APHIS.USDA.gov

**Keywords:** *Sirex noctilio*, parasitoids

## Abstract

Very little is presently known about the natural enemies and mortality factors associated with siricids (Hymenoptera: Siricidae) in the United States of America (USA), especially those that may directly affect the woodwasp, *Sirex noctilio* Fabricius (Hymenoptera: Siricidae). *S. noctilio* is an invasive woodwasp, is considered a major economic pest of pine, and has a severe effect on North American pine species planted in the Southern hemisphere. The mortality factors of siricid larvae were determined in three host species (*Pinus sylvestris*, *Pinus resinosa*, *and Pinus strobus*) from naturally infested trees in the northeastern USA. Siricid larvae were classified at the time of sampling as: (1) healthy, (2) parasitized by rhyssines (Hymenoptera: Ichneumonidae), (3) parasitized by *Ibalia* spp. (Hymenoptera: Ibaliidae), (4) parasitized by nematodes (Tylenchida: Neotylenchidae), and (5) dead from unknown causes. Combining data from the three host species, the average percentage of larvae that were healthy was 66%, 10% of the larvae were parasitized by rhyssines, 18% were parasitized by *Ibalia* spp., 1% were infected with unidentified nematodes, and about 5% of the larvae were dead in the galleries. Information from this study has important implications for understanding population regulation mechanisms in an invasive species, and will be critical for developing integrated pest management plans for *S. noctilio*.

## Introduction

In the northeastern United States there are more than half a dozen different native siricid species that utilize hardwoods and conifers (Smith and Schiff 2002). Recently an exotic woodwasp, *Sirex noctilio* Fabricius (Hymoneptera: Siricidae), was introduced into New York state. It kills living pine trees (Hoebeke et al. 2005) and has since been found in Pennsylvania, Michigan, Vermont, Ohio, and Connecticut. In its native Eurasian range, *S. noctilio* is considered a secondary pest. However, where the insect has invaded the southern hemisphere, it has been responsible for large economic losses to pine industries (Carnegie et al. 2006), primarily in commercial plantations of North American pine species. It is considered one of the most economically important pests of pine (Fernandez-Arhex and Corley 2005). In those areas where *S. noctilio* has become established there are few wood-boring insects attacking the trees, unlike the U.S., which has many native pine boring insect communities (Dodds et al. 2010). Many of the southern hemisphere countries introduced ichneumonid and nematode parasitoids as potential biological control organisms to combat *S. noctilio* (Cameron 1962, 1963, 1965; Bedding 1974). In the northeastern USA, hymenopterous parasitoids that are known to use siricids as hosts include the genera *Megarhyssa*, *Rhyssa*, and *Ibalia*. Nematodes in genus *Deladenus* (Tylenchida: Neotylenchidae) are also present in the northeast, and parasitize siricids, creating sterile females (Bedding 1978). The objective of this study was to assess the common mortality factors of siricid larvae, and to assess to what extent parasitism was occurring within *S. noctilio* colonized pine stands in the northeastern USA.

## Materials and Methods

### Sites and Host Trees.

Field sampling was conducted during December and January of 2007 and 2008 in pine stands that had known infestations of *S. noctilio*. Two different field sites were chosen for each host tree species, Scots pine, *Pinus sylvestris* L., red pine, *P. resinosa* Solander ex Alton, and white pine, *P. strobus* L. (Pinales: Pinaceae) per sampled year, for a total of four sites per host species. Field sites were unmanaged pine stands located in Oswego and Onondaga counties in central New York, USA. Five host trees were felled at each field site, resulting in a total of 20 trees felled per host species, and 60 trees felled between the two years of the study. The DBH of the trees chosen ranged from 6.5 – 9 inches. Host trees, symptomatic to a *S. noctilio* infestation, were chosen based on crown decay class, resin class, other insect activity (Coleoptera: Scolytidae, Cerambycidae, Buprestidae), and woodpecker activity. Trees were felled, and ten bolts of the same approximate length (51 cm) were chosen at random from the whole length of each tree. Bolts were labeled and brought back to the USDA laboratory in North Syracuse, NY.

### Splitting & Surveying.

In the laboratory, each bolt was split using a 4-ton electric log splitter (Ryobi Limited, USA Inc.). Bolts were split length-wise into pieces of approximately one-half inch or less width. All larvae were collected, identified to family, and tallied. Currently, there is no morphological key for identifying siricid larvae, and molecular techniques were not developed and available at the time that this study took place. Therefore, all horntail larvae were identified to family only.

Dead siricid larvae were tallied, and typically either had a completely blackened cuticle, or were covered in a white or green fungus. Siricid larvae that were parasitized by rhyssines (Ichnuemonidae: Rhyssinae) were tallied during examination of the siricid gallery because they are ectoparasitoids, and easily detectable on a siricid larva. All other live siricid larvae were placed individually in vials sorted by tree, and dissected to determine if they had been parasitized by *Ibalia* spp. (Ibaliidae) or parasitic nematodes (*Deladenus* spp.).

The remaining siricid larvae that were not dead or parasitized were considered to be healthy at the time of sampling. In addition, 20 siricid galleries, chosen at random from several different log billets from several trees of each tree species, were measured (mm) from the center of the long axis of the gallery to the edge of the bark layer in order to determine if gallery depth has an effect on parasitism.

### Data analysis.

Data presented as the overall percentage of larvae in each mortality category (i.e., healthy, dead, parasitized, etc.) were estimated using the following formula, modeled after Fukuda and Hijii (1996):
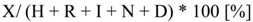

where “X” is the number in the mortality category being determined (i.e., % parasitism by rhyssines), “H” denotes the number of healthy larvae found throughout the 200 log bolt samples per host species, “R” denotes the number of rhyssine larvae, “I” denotes the number of *Ibalia* spp. larvae, “N” denotes the number of siricid larvae that had nematodes inside the body cavity, and “D” denotes the number of siricid larvae found dead from other or unknown causes in the galleries.

An Oneway ANOVA was used to analyze the arcsin square root transformation of the proportion of siricid larvae found in each mortality category by host species. Each tree was considered a replicate. An Oneway ANOVA was also used to analyze the distance of siricid galleries to the bark edge by host species, with each gallery treated as a replicate. A Tukey-Kramer HSD was used for analysis among treatments for statistically significant results (JMP 8.0, SAS Institute Inc., 2008).

## Results

Because the trees sampled were symptomatic to *S. noctilio* infestation, it is presumed that most of the larvae sampled were *S. noctilio*. However, molecular confirmations were not used in identification of the larvae, and so they are referred to as siricids because of the possibility of native species being present. Among the three different host species, a total of 2,125 siricid larvae were found in the sampled Scots pine trees, 443 in the red pine, and 76 in the white pine. Of the three host species combined, there was a total of 2,644 siricid larvae found developing in the logs. Out of the 2,644 larvae, 1,743 were found healthy, 264 larvae were parasitized by Ichneumonidae, 484 were parasitized by Ibaliidae, 30 were parasitized by nematodes, and 123 larvae were found dead at the time of sampling. Parasitism by *Ibalia* spp. was the greatest mortality factor (18%) on siricid larvae in this study when all three host species data were combined. The total and percentage of larvae in each fate category per host species can be found in Table 1.

**Table 1.**
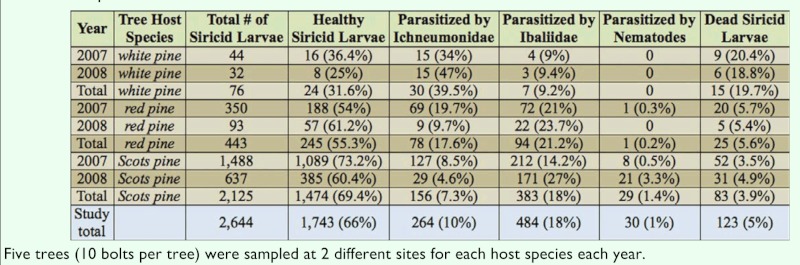
The total number (and percentage) of larvae found in each mortality category from 200 billets out of the 20 trees of each host species that were examined.

Although the proportion of larvae that appeared healthy varied from 31.6% in white pine to 69.4% in Scots pine, there was no significant difference in the proportion of healthy larvae found among host species (*F*_2, 58_ = 1.46; *P* = 0.24). Proportions of larvae parasitized by *Ibalia* spp. (*F*_2, 58_ = 1.54; *P* = 0.22) and rhyssines (*F*_2, 58_ = 2.21; *P* = 0.12) also did not differ significantly by host species. There were, however, significant differences detected among tree host species for siricid larvae parasitized by nematodes (*F*_2, 58_ = 5.71; *P* = 0.006). More nematode parasitism was detected in siricid larvae from Scots pine (0.014 ± 0.003) than larvae from white pine (0 ± 0.003) and red pine (0.002 ± 0.003). The nematodes that were found associated with the siricids in this study were not identified using molecular techniques, but are presumed to be an endemic strain of *Beddingia siricidicola* introduced with *S. noctilio* (Yu et al 2009; Williams et al. 2010).

**Figure 1.**
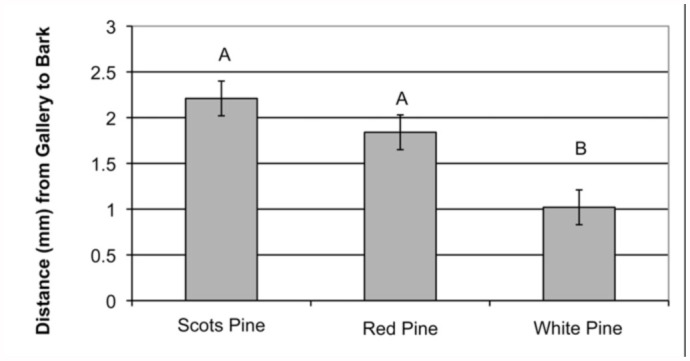
Siricid galleries were found significantly closer (*F*_2, 57_ = 9.74; *P* = 0.0002) to the edge of the bark layer in white pine (1.03 ± 0.19) than in red pine ( 1.85 ± 0.19) or Scots pine (2.21 ± 0.19). Small bars represent SEM. Capitalized letters represent treatment groups that are statistically different from one another. High quality figures are available online.

There were no host species differences associated with the number of siricid larvae that were found dead from unidentified causes *(F*2, 58 = 2.01; *P* = 0.14). Additionally, we found that there was a significantly shorter distance in the average siricid gallery to the bark edge in white pine (1.03 ± 0.19 mm) compared to both Scots pine (2.21 ± 0.19 mm) and red pine (1.85 ± 0.19 mm) (*F*2,_58_ = 9.74; *P* = 0.0002), but no difference between Scots and red pine (Fig. 1).

The greatest mortality factor for siricids in Scots pine is parasitism by *Ibalia* spp. (18 %) (*F*_3, 80_ = 7.93; *P* = 0.0001). There were no statistically significant differences in mortality factors found in red pine (*F*_3, 76_ = 2.65; *P* = 0.0548). In white pine, there was no significant difference between the level of parasitism by rhyssines and larvae that were found dead in the galleries, though rhyssine parasitism was more frequent than parasitism by *Ibalia* and nematodes (*F*_3, 76_ = 5.11; *P* = 0.0028).

## Discussion

For the management of *S. noctilio*, it is important to understand the effect natural enemies currently have in the North American siricid complex. There were far more siricid larvae in the infested Scots pine trees sampled (2,125) than in red pine (443) or white pine (76). This finding may be attributed to differences in host volatile emissions that alter attractiveness to ovipositing females. Boröczky et al. (2012) found that Scots pine are releasing quantitative and qualitatively different volatile blends, rendering them more attractive to *S. noctilio* females than other hosts. Similarly, Dodds et al. (2010) found that Scots pine exhibit higher level basal area loss from *S. noctilio* colonization over other host species in the northeastern U.S.

There has been a significant amount of work done elucidating the behavior and ecology of parasitoids of siricids (Rawlings 1952, 1953; Cameron 1965; Spradberry 1970a, 1970b; Spradbery and Ratkowsky 1974; Taylor 1978; Fernandez-Arhex and Corley 2005). Despite this work, limited data are published on the natural parasitism rates in North American forests. Most of the reports of North American parasitoids are laboratory and artificial rearing studies (Taylor 1967; Spradbery 1970; Fukuda and Hijii 1996). There is currently limited data on the impact natural enemies have where they are not manipulated for biological control strategies. One research group, Long et al (2009), described results on parasitism of *S. noctilio* from six Scots pine. The results documented here support the preliminary findings by Long et al. (2009). The authors reported a percentage of parasitism (20.5%) by *Ibalia leucospoides* in Scots pine similar to what we describe (18%). Another research group, (Fukuda and Hijii 1996), describe parasitism in *Sirex nitobei* M. from two naturally infested pine trees. The authors found a combined parasitism rate of 60% from *Megarhyssa praece liens* and *I. leucospoides*. Most of the available work on parasitoids of siricids investigates the testing of North American species in southern hemisphere countries to aid in the control of *S. noctilio* (Rawlings 1951, 1952, 1953; Cameron 1962, 1963, 1965; Spradbery 1970a, 1970b, 1974; Taylor 1978; Fernandez-Arhex 2005).

Spradbery (1970) reported that *Rhyssa persuasoria* was specifically keying into the fungus-produced odor in the frass nearest the developing larva. Additionally, Long et al. (2009) found that there was significantly less parasitism of siricids by *Ibalia* as tree diameter increased. Thus, it seems likely that to the closer the gallery to the bark surface, the more attractive and accessible it would be for parasitism by natural enemies, specifically the hymenopteran parasitoids. In this study, we found galleries were closer to the bark surface in white pine (Figure 1), with more observed parasitism by both hymenopteran families combined in white pine (48.7%) than in red pine (38.8%) and Scots pine (25.3%) (Table 1).

The nematode *Deladenus siricidicola* B. has been used extensively in the southern hemisphere to help control *S. noctilio* (Bedding 1979; Haugen 1990; Lopez et al. 2004; Tribe and Cillié 2004: Hurley et al. 2008). There are several species of naturally occurring *Deladenus* nematodes that sterilize woodwasps, but *D. siricidicola* was specifically selected because of its high parasitism rate, and because of its specificity to *S. noctilio* and its symbiotic fungus (*Amylostereum areolatum* B.), which is utilized by the nematode in its fungus-feeding form (Bedding and Akhurst 1978; Bedding 1979). We first documented an endemic strain of *D. siricidicola* associated with trap-caught *S. noctilio* in New York state in 2006 (unpublished data), and it has also been confirmed in Canada (Yu et al. 2009). Nematodes were only found in 1% of the larvae sampled in this study, but it is critical to understand the dynamics and role the endemic nematodes have in the USA before a release of a biological control stain that could potentially complicate the management of *S. noctilio*. Further studies are currently being conducted in this area.

This information is important because it sheds light on the impact natural enemies may have on the local populations of siricids in three different host species, and it provides background information for North American management programs of *S. noctilio*.
